# Characterization of Polysulfides, Polysulfanes, and Other Unique Species in the Reaction between GSNO and H_2_S

**DOI:** 10.3390/molecules24173090

**Published:** 2019-08-26

**Authors:** Murugaeson R Kumar, Patrick J Farmer

**Affiliations:** Department of Chemistry and Biochemistry, Baylor University, Waco, TX 76798, USA

**Keywords:** hydrogen sulfide, S-nitroso glutathione, polysulfanes, sulfides, sulfenic acids, high-resolution mass spectrometry, dimedone, iodoacetamide, vinylcyclopropane

## Abstract

Glutathione-based products, GS_n_X, of the reaction of hydrogen sulfide, H_2_S, S-nitroso glutathione, and GSNO, at varied stoichiometries have been analyzed by liquid chromatography high-resolution mass spectrometry (LC-HRMS) and chemical trapping experiments. A wide variety of glutathione-based species with catenated sulfur chains have been identified including sulfanes (GSS_n_G), sulfides (GSS_n_H), and sulfenic acids (GS_n_OH); sulfinic (GS_n_O_2_H) and sulfonic (GS_n_O_3_H) acids are also seen in reactions exposed to air. The presence of each species of GS_n_X within the original reaction mixtures was confirmed using Single Ion Chromatograms (SICs), to demonstrate the separation on the LC column, and given approximate quantification by the peak area of the SIC. Further, confirmation for different GS_n_X families was obtained by trapping with species-specific reagents. Several unique GS_n_X families have been characterized, including bridging mixed di- and tetra-valent polysulfanes and internal trithionitrates (GSNHS_n_H) with polysulfane branches. Competitive trapping experiments suggest that the polysulfane chains are formed via the intermediacy of sulfenic acid species, GSS_n_OH. In the presence of radical trap vinylcyclopropane (VCP) the relative distributions of polysulfane speciation are relatively unaffected, suggesting that radical coupling is not a dominant pathway. Therefore, we suggest polysulfane catenation occurs via reaction of sulfides with sulfenic acids.

## 1. Introduction

In the last decade, hydrogen sulfide (H_2_S) has been identified as an important gasotransmitter [1], analogous to NO, CO, and other small molecules that act as signal transducers in mammalian physiology [2]. In particular, H_2_S and other sulfur-releasing compounds are found to mimic numerous physiological effects of NO related to cardiovascular function [3]; a number of studies suggest an interesting synergism between the two small gasses [4,5]. Thus, there is speculation that vasorelaxation effects may be induced through some biotransformation of H_2_S and NO resulting in polysulfide (RSS_n_H) [6,7], polysulfane (RSS_n_R) [8,9], and S-nitroso (RSNO) [10] species. All of these derivatives have been shown to be endogenously formed in cells, but their generation and activities are still much debated [11].

Distinct biosynthetic pathways of sulfur-concatenated species such as polysulfides have been identified in the last five years. Kimura found that 3-mercaptopyruvate sulfurtransferase and cysteine aminotransferase localized to a vascular endothelium produce H_2_S and persulfides H_2_S_n_ (n = 1–3) [12], as well as cysteine- and glutathione-persulfides [13]. Akaike et al. identified cysteinyl-tRNA synthetases that generate cysteine polysulfides (CysSS_n_H) which are integrated into proteins during translation [14,15]. These polysulfides were shown to be superior nucleophiles and reductants, and capable of regulating electrophilic cell signaling mediated by 8-nitroguanosine 3′,5′-cyclic monophosphate [16,17]. A recent report described the direct activation of CGMP-dependent protein kinase by polysulfides independent of known biological pathways [18]. There are numerous synthetic routes to sulfur-concatenated species, including the reaction of thiols with elemental sulfur [19], but the mechanisms for biological polysulfide generation are still obscure [20].

Polysulfides and polysulfide oxides are generated by outer-sphere oxidation of H_2_S with biologically relevant oxidants such as metmyoglobin, hydroxycobalamin and MP-11 (a heme-adduct fragment of cytochrome c) through the initial generation of small oxoacids of sulfur and SOS [21,22]. We proposed that small oxoacids of sulfur, SOS, such as HSOH and HOSOH may be biologically relevant precursors to sulfur-concatenated species through condensation reactions with thiols. In this report, we demonstrate the intermediacy of such sulfur-oxides in the generation of polysulfides in a reaction of relevance to cardiovascular signaling.

GSNO is an active biological nitrosating agent as well as a source of NO [23,24,25,26,27]; it has been used to model the bioreactivity of protein S-nitroso species. Several researcher groups have examined the reaction of GSNO with H_2_S, which primarily produces NO, and which has been suggested as the likely pathway of H_2_S-induced vasodilation [28]. But this reaction also produces a myriad of other species that may generate a similar signaling response, e.g., SSNO^−^, HSNO, and HNO [8,11,28,29,30,31]. The detailed mechanism and speciation of products from this reaction remain quite contentious [29,31].

**Scheme 1:**
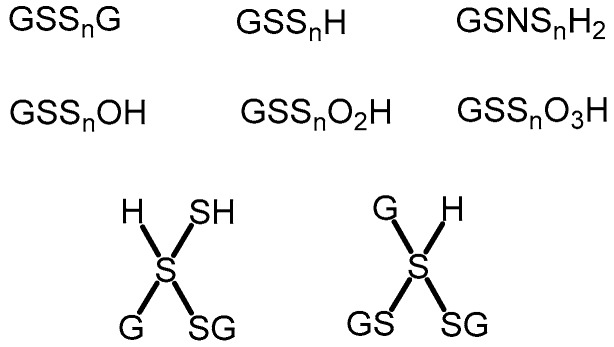
Species generated in the reaction between GSNO and Na_2_S.

We too have previously examined the reaction of GSNO and H_2_S, and focused mainly on the nitrogenic product speciation and quantification [32]. In this report, we focus on the large menagerie of unusual glutathione-based products, GS_n_X, from this reaction, which includes families of polysulfides and polysulfanes (Scheme 1), as well as bridging polysulfanes analogous to thioacetals of sulfinyl S(IV) species and internal trithionitrates (GSNHS_n_H) with polysulfane branches.

**Scheme 2:**
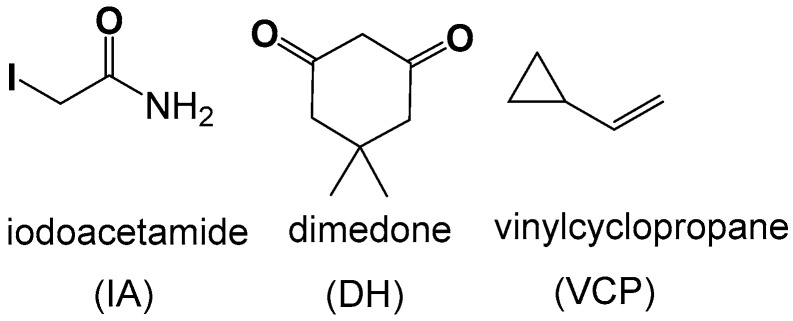
Structures of electrophilic, nucleophilic and radical traps used in this study.

These various species were characterized by Orbitrap liquid chromatography-high resolution mass spectrometry (LC-HRMS), using selective traps shown in Scheme 2. The S-H selective trap iodoacetamide (ICH_2_CONH_2_ or IA) alkylates thiols generating stable thioethers which are readily characterized by HDMS. The S-OH selective trap dimedone (DH) which reacts with sulfenic acids in proteins [33]; it and its derivatives have been widely used to characterize S oxidation biological milieus [34,35,36]. The radical trap vinylcyclopropane (VCP) is used to assess the possible involvement of S-based radicals in the formation of polysulfane chains. Although exact quantification of the whole menagerie of GS_n_X species is difficult, some mechanistic insight is provided by the distribution patterns of the various species under different conditions.

## 2. Results and Discussion

### 2.1. Reaction Methodology and Overview

In a typical experiment, stoichiometric concentrations of GSNO and Na_2_S, which generates H_2_S/HS^−^ within the reaction mixture, were reacted in neutral buffer solutions. The concentrations of GSNO and Na_2_S used followed those used in previous reports [32,37]. The combination of these reagents produces a yellow solution indicative of the presence of the thionitrites SNO^−^ and SSNO^−^, which fades over 15 min [8]. The final reaction mixtures were then analyzed using a high-sensitivity LC-HRMS instrument that allows for a mass resolution of ~30,000 m/Δm at m/z = 400. Selected ion chromatograms (SICs) were determined which characterize the elution profile of a single chosen species with a specific mass-to-charge ratio within a complex LC-HRMS trace.

### 2.2. Characterization of Glutathione Polysulfanes (GSS_n_SG)

**Figure 1:**
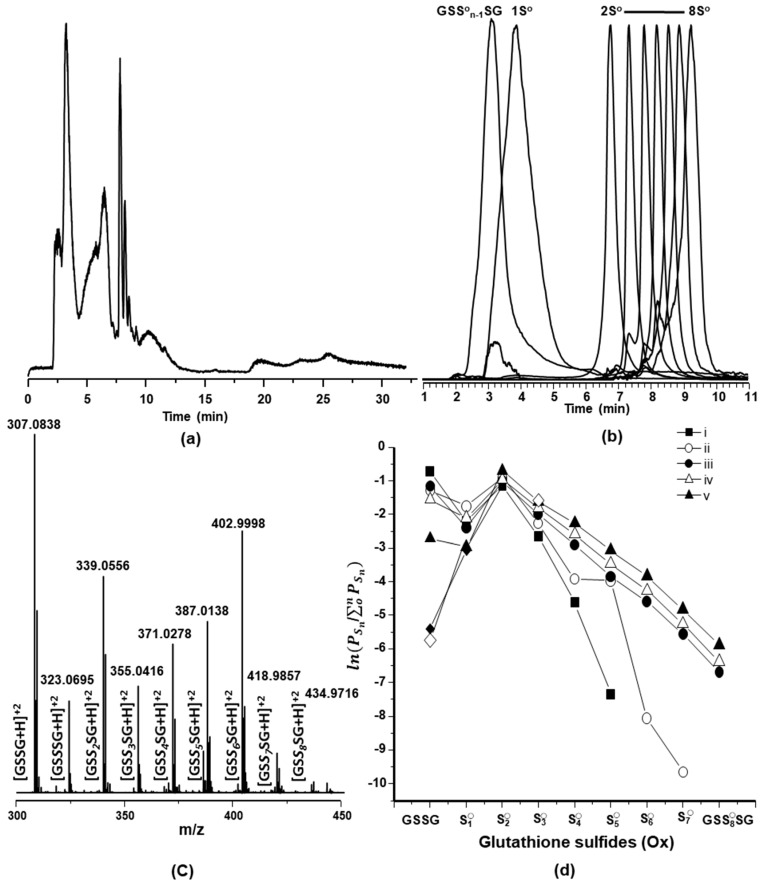
Liquid chromatography-high resolution mass spectrometry **(**LC-HRMS) analysis of oxidized glutathione polysulfane species formed in a 1:5 reaction of GSNO with Na_2_S at pH 7, with 0.1% formic acid-acetonitrile as a carrier stream. (**a**) The total ion LC spectra. (**b**) Normalized selective ion chromatograms (SICs) showing a series of GSS_n_SG polysulfanes, n = 0–8, and the separation times of each within the overall LC spectra. (**c**) Region of the total ion mass spectra showing peaks assigned as shown, to dications. (**d**) Logarithmic representations of speciation concentrations calculated from the peak area of the respective species in the LC-HRMS spectral analysis of oxidized glutathione polysulfanes in the reaction of one equivalent of GSNO with increasing equivalents of Na_2_S under aerobic condition: (i) 0.25, (ii) 0.5, (iii) 1, (iv) 2.5 and (v) 5 equivalents of Na_2_S.

The initial glutathione-based products observed by LC-HRMS were the oxidized polysulfanes, GSS_n_SG (n = 0 to 8), which have been previously shown to be produced in these reactions [38]. Figure 1 shows a typical LC trace (Figure 1a) with individual SIC peaks (Figure 1b) obtained by refining a total ion mass spectrum to a unique m/z value attributable to each GSS_n_SG species (Figure 1c). The elution profiles of these species occurred within the first 10 min of separation, both the separation and sequential LC spacing of the individual SIC peaks confirm that each species was present in the original reaction mixture. Notably better LC separation was obtained using formic acid/methanol and formic acid/acetonitrile (Supplementary Materials S1) as carrier streams, and both separation methods identified multiple SIC peaks, indicating the separation of individual species present within the original reaction product mixture. The presence of the GSS_n_SG species was also confirmed by MS/MS studies which show expected fragmentation patterns (Supplementary Materials S2).

Analysis of the peak area of individual GSS_n_SG SICs allows the comparison of relative concentrations of the individual species, with the assumption that the relative ionization efficiencies are the same within a family of similar glutathione species. The SIC peak areas for GSS_n_SG species were determined and a logarithmic plot is shown in Figure 1d, for a series of reactions in which the stoichiometry of Na_2_S to GSNO varied from 0.25 to 5 under aerobic conditions. The logarithmic plot better illustrates the relative distributions of both higher and lower concentrations of polysulfane species generated in these reactions.

Several control experiments were carried out to assess the effect of pH, temperature, and sulfide/sulfane source on the product distributions (Supplementary Materials S3–S9). Similar distribution profiles were observed at pH 7 and 10 (Figure S3), at 25 °C and 35 °C (Figure S4), as well as ones using gaseous H_2_S instead of solid Na_2_S (Figure S5). GSSG reacts with H_2_S generating GSS_n_SG species with n = 1–4. As was previously reported, elemental sulfur, S^0^, is incorporated into both GSSG and GSH forming polysulfanes (Figure S6), but no reaction is seen with GSNO. The presence of S^0^ in situ during reactions of GSNO and Na_2_S had little overall effect on product distribution (Supplementary Materials S7). Thiosulfate reacts with GSH to form GSS_n_SG species with n = 2–5 (Supplementary Materials S8), and the addition of thiosulfate to reactions of GSNO and Na_2_S does affect the resulting GSS_n_SG distribution (Supplementary Materials S9).

### 2.3. Glutathione Polysulfides GSS_n_H and Alkylated Derivatives

**Figure 2:**
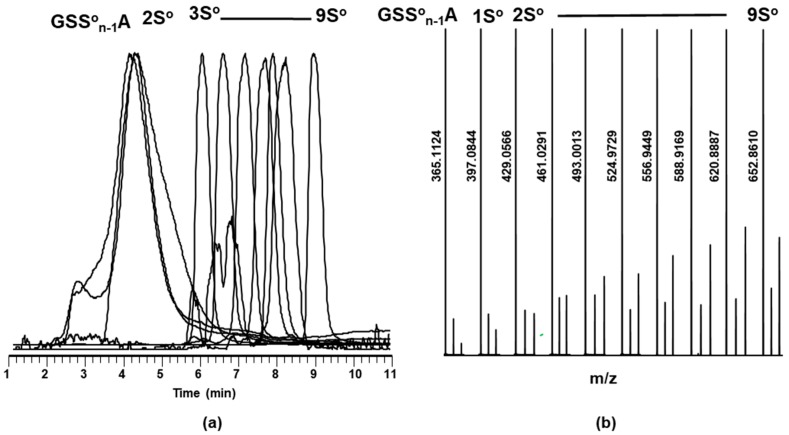
(**a**) Individual SICs glutathione polysulfane acetamide adducts obtained, RSS_n_CH_2_CONH_2_ (n = 0–9) from LC/MS analysis of GSNO (1 mM) with an Na_2_S (1 mM) reaction in the presence of IA (5 mM) in iP buffer pH 7 and (**b**) their corresponding SIC MS.

The presence of reduced polysulfide derivatives was then investigated. Only very small signals for reduced polysulfide anions, GSS_n_^−^, were observable in negative ion mode; much better signals were obtained for the protonated GSS_n_H in positive ion mode, but only a limited number were detected, (Supplementary Materials S10 and S11). A larger range of polysulfides was identified using the S-H selective trap IA, characterization as alkylated polysulfides GSS_n_A (where A = CH_2_CONH_2,_ and n = 0 to 9), Equation (1). As shown in Figure 2b, the normalized individual SIC mass spectra show the expected increase of the (m + 2) isotopomer over the series, due to the increase in ^34^S which has a natural abundance of 4.29%.

GSS_n_H + IA → GSS_n_A + HI (1)

**Figure 3:**
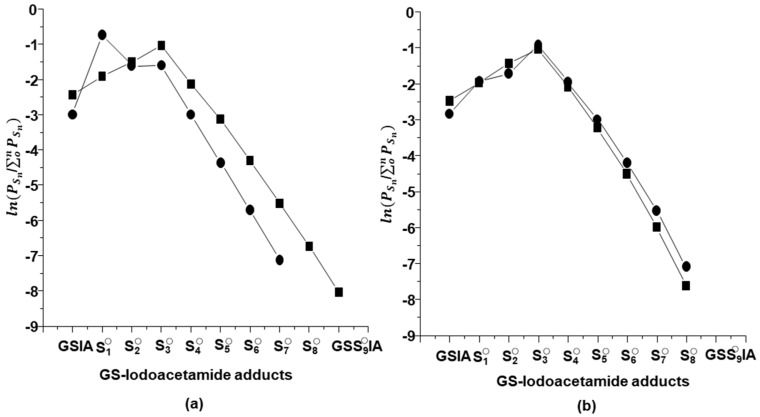
Logarithmic relative distribution of GSS_n_A adducts in (**a**) aerobic and (**b**) anaerobic reactions between GSNO and Na_2_S (1:1) with IA present at the beginning (filled circle) or added after 1 hr initiation (filled square) as calculated from the peak area of their respective SICs.

This mild alkylation method allows comparison of polysulfane formation under different experimental conditions by trapping in situ or at time periods after the reaction, and under aerobic or anaerobic conditions. As shown in Figure 3, the relative distributions of polysulfanes trapped in situ are remarkably similar under both aerobic and anaerobic conditions, and between in situ alkylations and those following reaction terminations. The one difference in distribution occurs under aerobic conditions, likely due to the oxidation of polysulfanes by dioxygen (Equation (2)) yielding S-oxygenated products, as below.

### 2.4. Characterization of Glutathione Oxoacids, Sulfenic, Sulfinic, and Sulfonic

**Figure 4:**
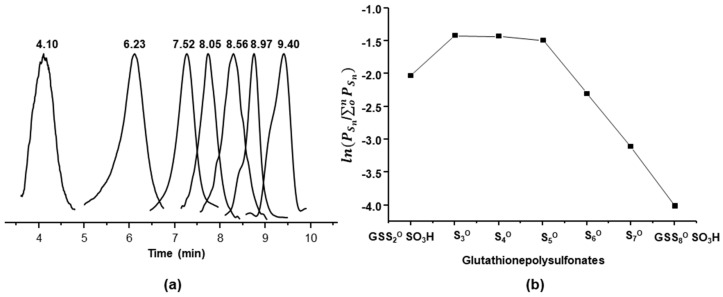
Normalized SIC (**a**) mass spectra of oxidized glutathione polythiosulfonates, GSS_n_SO_3_H, generated in the aerobic reaction of GSNO (1 mM) with Na_2_S (1 mM) in IP buffer at pH 7 and (**b**) relative distribution as calculated from the peak area of the SICs.

The observed effect of air on the distribution of polysulfides suggested that oxygenated species would be found in aerobic reaction mixtures. Indeed, SICs of glutathione polysulfane thiosulfonates, GSS_n_SO_3_H, with n = 2–8 were identified in aerobic reactions, Figure 4 (LC-MSMS spectra are given in Figure S12). The smaller polysulfane thiosulfonates, e.g., GSSO_3_H, with n = 0–2 were only observed in negative ion mode. The low signals of the smaller thiosulfonates, suggest that these GSS_n_SO_3_H species were mainly derived by reactions of the longer polysulfanes with dioxygen. Weak LC-HRMS peaks corresponding to the sulfinic derivatives, GSSO_2_H, are observable only in negative ion mode at very low signal-to-noise ratios in aerobic reaction mixtures (Figure S13, LC-MSMS Figure S14).

GSS_n_H + O_2_ + H_2_O → GSS_n−1_O_y_H (2)

**Figure 5:**
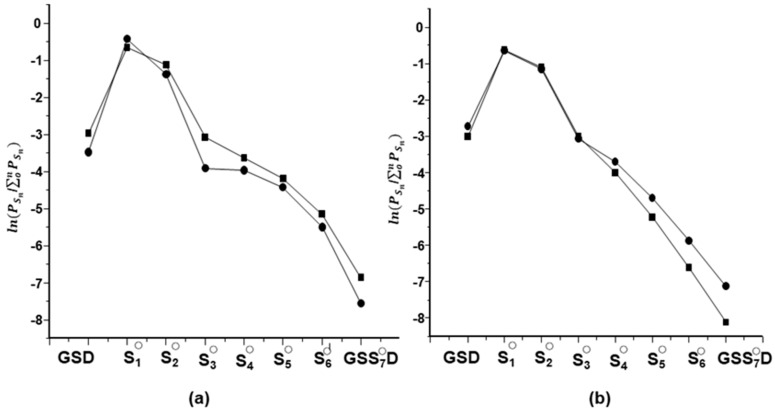
Relative yields of GSS_n_D adducts in (**a**) aerobic and (**b**) anaerobic reactions between GSNO and Na_2_S (1:5 mM) with DH present in situ (squares in **a** & **b**) or added after 1 h initiation (circles in **a** & **b**) as calculated from the peak area of the SICs.

The possibility of sulfenic products such as GSS_n_OH, was assessed using the trapping agent dimedone DH, which selectively attacks the electrophilic sulfenic acid moiety to yield stable thioethers, such as GSS_n_D, as in Equation (3). Dimedone-trapped GSS_n_D were observed in equal distributions and quantities under both aerobic and anaerobic conditions as shown in Figure 5, therefore these species are not generated by reaction with dioxygen. These sulfenic acids were also found in similar distributions when trapped by DH in situ or 1 h after reaction initiation. Significantly, the smallest derivative GSD was seen under all conditions, implying that the cystenic sulfur of GSNO is directly formed during the reaction with H_2_S; control experiments show that there was no nucleophilic attack of DH, on the cystenic sulfur of GSH, GSSG, or GSNO under our experimental conditions. Using similar trapping methods, we found GSD was the major product from the reduction of GSNO with ascorbate and other reductants (Figure S15) [32].

GSS_n_OH + D → GSS_n_D + H_2_O (3)

### 2.5. Mixed Valence Sulfide Products

**Scheme 3:**
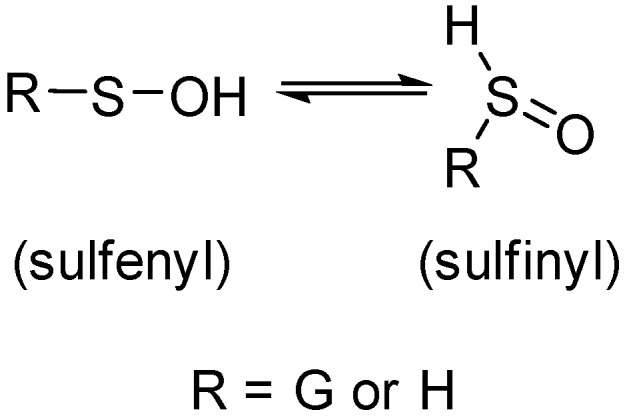
Sulfenic acid tautomers.

In a recent report, we demonstrated that the sulfenyl and sulfinyl tautomeric forms, Scheme 3, of glutathione sulfenic acids can be trapped by various C-type nucleophilic traps like dimedone, 1-trimethylsiloxycyclohexene, and cyanide [39]. The dual reactivity of sulfenic acid as nucleophile and electrophile towards trapping agents produced unique products that are well characterized by LC/HDMS studies. The prevalence of sulfenic products in these reactions of GSNO with H_2_S suggested a similar formation of mixed di- and tetra-valent species, sulfanedithiols which are analogous to thioacetals, Equations (4) and (5).

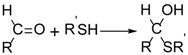
(4)

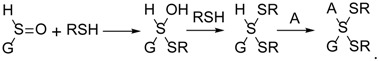
(5)

**Figure 6:**
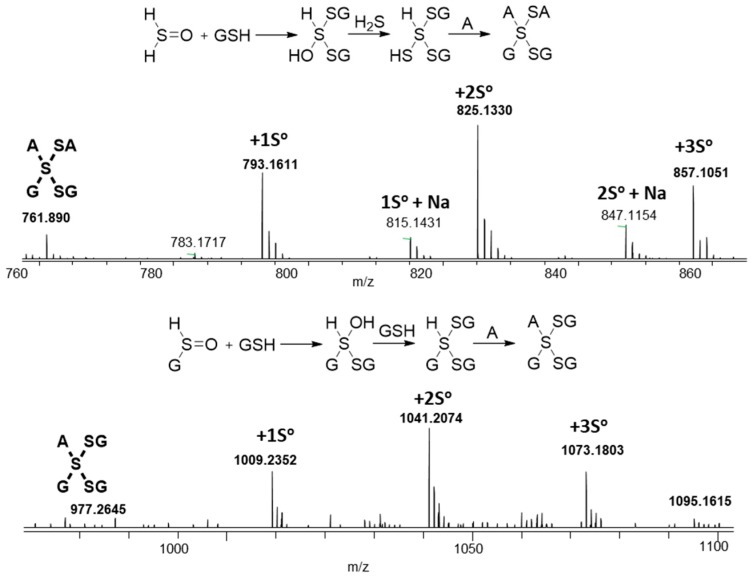
Mass spectra of sulfanedithiols derived from sulfinyls of (H_2_SO) and GS(O)H in the reaction of GSNO (1 mM) and Na_2_S (1 mM) in the presence of IA (5 mM) in iP buffer, pH 7.

A number of polysulfane products with the apparent bridging of S(IV) sulfinyl centers were also identified, albeit in low yields. Figure 6 displays two families of such species, one with m/z corresponding to two glutathione and two acetamide groups attached to 3–6 sulfur atoms; the second with three glutathione and one acetamide attached to 3–6 sulfur atoms. As in Scheme 3, these species may result from thiol addition to sulfinyl tautomers. Although rarely characterized, a recent density functional theory study [40] has shown that inclusion of water solvation dramatically lowers the energy of the S(IV)sulfinyl tautomers, suggesting that mixed-valence sulfide species may be more prevalent in aqueous solution. Alternatively, these bridging S(IV) species may be generated from equilibrating insertion reactions of a trisulfane species with a disulfide bond, as seen in Equation (6), and also predicted by gas-phase calculation to have a higher reaction enthalpy than S-S bond dissociation [41].

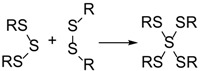
(6)

### 2.6. N-Containing GSX

It is well documented that HNO reacts with GSH to form a thiohydroxamic acid, GSNHOH, which rearranges to yield a stable sulfonamide, GSONH_2_ [42]; this stable sulfonamide has been used as an analytical marker for the presence of free HNO in cells [43]. Indeed, both the reaction of GSH with HNO donor Angeli’s Salt and the reaction of GSNO with NaBH_4_, result in the observance of a sulfonamide in MS spectra [32]. But notably, no sulfonamides were observed in the reactions of GSNO with H_2_S, which suggests that no free HNO was formed in these reactions.

**Scheme 4:**
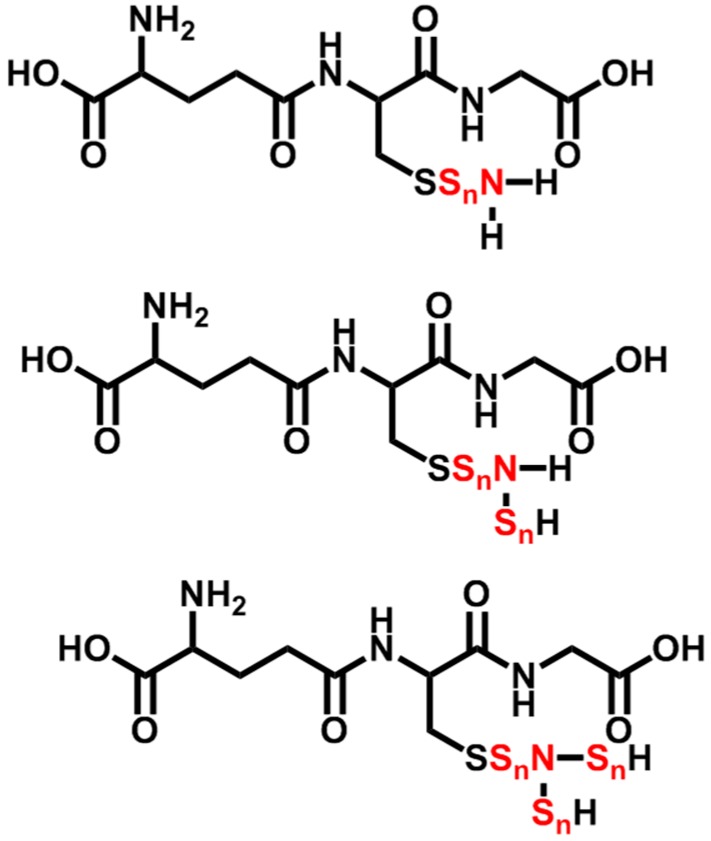
Structural isomers of trithionitrates.

**Figure 7:**
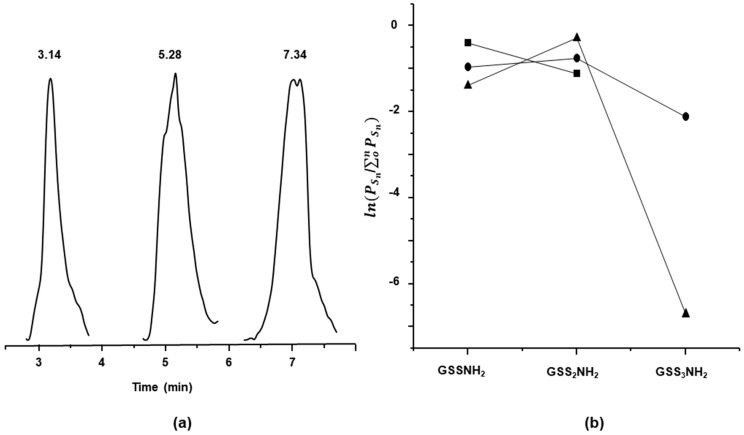
(**a**) Normalized SICs of glutathione sulfonamide GSS_n_NH_2_ in the reaction of GSNO (1 mM) with Na_2_S in iP buffer at pH 7 and (**b**) relative distribution as calculated from the peak area of the SICs for reactions at Na_2_S ratios of (square) 0.25, (circle) 0.5, (rectangle), 1 mM.

**Figure 8:**
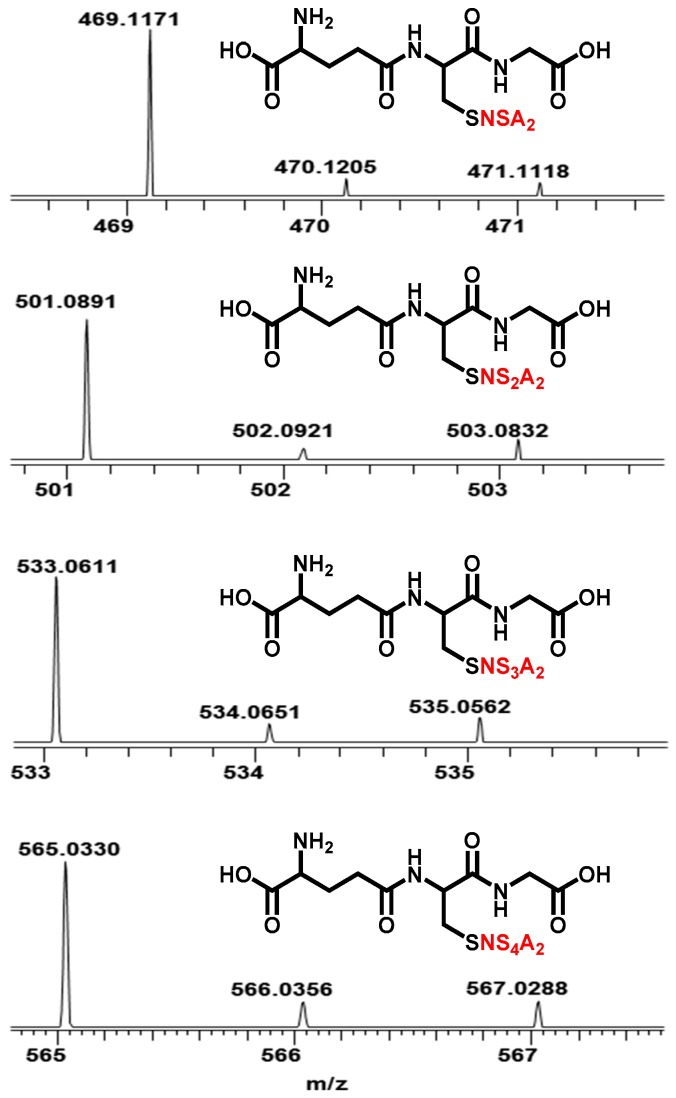
MS of GSS_n_NA_2_ in the reaction GSNO (1 mM) and Na_2_S (1 mM) in the presence of IA (5 mM) in iP buffer, pH 7.

A short series of N-containing polysulfanes GSS_n_NH_2_, Scheme 4, with a range of polysulfanes, from n = 1–3, were identified under anaerobic conditions, Figure 7 (LC-MSMS Figure S16). These species are only observed in reactions with low sulfide concentrations, at ratios of 1:0.25 and 1:1 of GSNO:Na_2_S. No GSNH_2_ was seen, which precludes direct reduction of the parent GSNO. A plausible pathway to one isomeric form of GSS_n_NH_2_ is by sequential nucleophilic attack of HS^−^ on the nitrogen of GSNO followed by dehydration, yielding trithionitrites, as seen in Equation (7). In agreement with this suggestion, analogous alkylated versions, GSNS_n_A_2_, were identified in reactions run with the S-H trap IA, Equation (8), as shown in Figure 8 (LC-MSMS Figure S17).

GSNO + HS^−^ → GSN(OH)SH + HS^−^ → GSN(SH)_2_ + H_2_O (7)

GSN(SH)_2_ + IA → GSN(SA)_2_ (8)

### 2.7. Competitive Trapping Experiments

**Figure 9:**
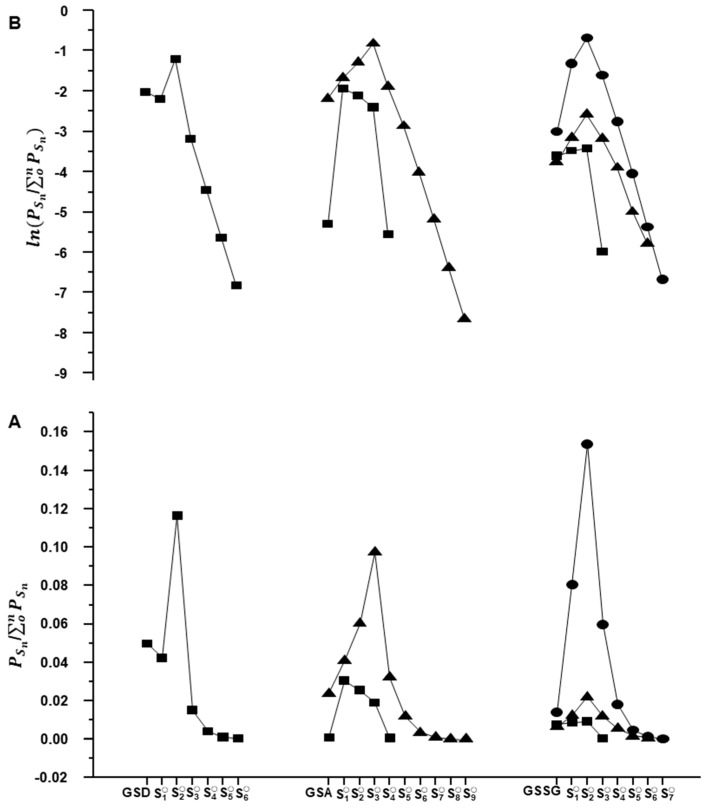
Relative distributions (**A**) and natural logarithm of relative distributions (**B**) of DH trapped GSS_n_D, acetamide trapped GSS_n_A, and oxidized GSS_n_G species determined from reactions of GSNO (1 mM) with Na_2_S (1 mM): (Squares) in the presence of DH (50 mM) and IA (50 mM); (triangles) in the presence of IA only; (circles) in the absence of DH and IA in iP buffer at pH 7.

The relative production (by peak area) of the main GSX products (GSS_n_SG, GSS_n_H, and GSS_n_OH) were assessed in reactions run in the presence and absence of DH and IA. To analyze the results of these experiments, the total ion count of all three main families of products were used in the distribution plots, shown as both the raw data as well as the natural log $\ln(P_{S_{n}}/\sum_{o}^{n}P_{S_{n}})$ in Figure 9. As shown, the distributions of the three species follow similar patterns when exposed to only one trapping agent, with maxima at for GS_n_SOH and GSS_n_SG at n = 2, GSS_n_A at n = 3.

**Scheme 5:**
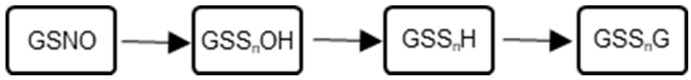
Proposed sequence of speciation

In reactions run in the presence of IA, the generation of GSS_n_SG is greatly attenuated implying the polysulfide precedes the oxidize polysulfanes. In reactions run with both DH and IA traps, both GSS_n_SG and GSS_n_A species are dramatically diminished, implying that the sulfenates act as precursors to reduced polysulfides, shown in Scheme 5.

We have previously shown that small oxoacids of sulfur, SOS, such as HSOH and HOSOH can be trapped during the aqueous oxidations of H_2_S [21]. Analogous trapped species (AS_n_D, AS_n_A, and DS_n_D) are also found in all reactions of GSNO with H_2_S, but at very low relative concentrations. Therefore, we hypothesize that the glutathione polysulfanes are generated by stepwise additions of these sulfenates, as in Equations (9) and (10).

GSS_n_H + HSOH → GSS_n_H + H_2_O (9)

GSS_n_H + HOSOH → GSS_n_OH + H_2_O (10)

### 2.8. Role of S Radicals in Polysulfane Formation

The involvement of radical coupling in polysulfane formation have been noted in LiS batteries [44], but relatively little evidence of radical coupling exists in the biological literature [45]. We posited that a radical trap should interfere with the range and relative distribution of GSX polysulfanes if such species are formed via S radical coupling. The most widely used radical traps are those that form stable nitroxyl adducts, like 5,5-dimethyl-1-pyrroline N-oxide (DMPO) and its phosphorylated version 5-diethoxyphosphoryl-5-methyl-1-pyrroline N-oxide (DEPMPO) [46]. However, these traps are also susceptible to nucleophilic and reductive reactions, which make them problematic in GSNO/H_2_S reactions. Our solution was to use a so-called radical clock, vinylcyclopropane (VCP), which reacts with radical species to generate ring-opened products, seen in Equation (11) [47], but is unreactive with nucleophiles or reductants.

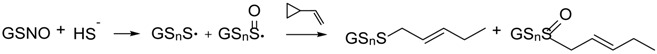
(11)

**Figure 10:**
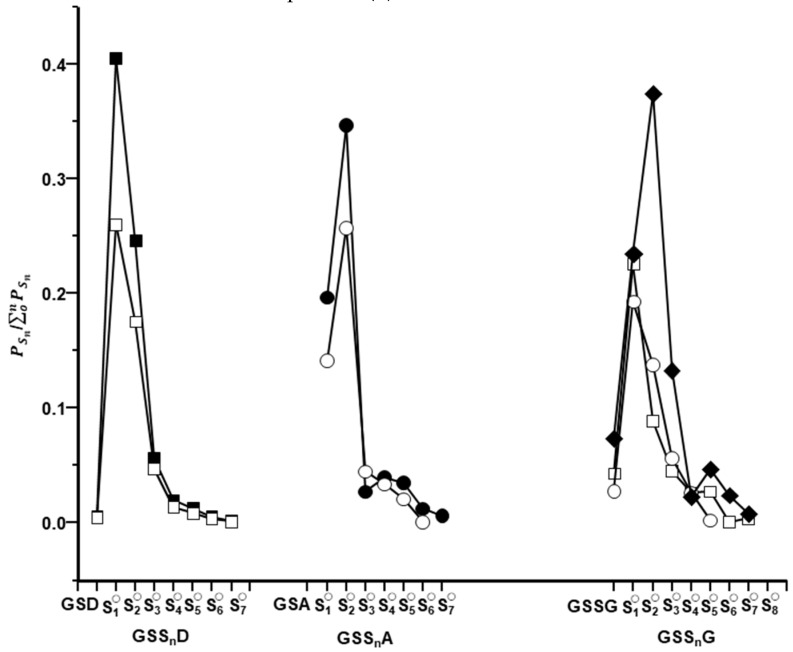
Relative distributions of DH trapped GSS_n_D, acetamide trapped GSS_n_A, and oxidized GSS_n_G species determined from LCMS study of reaction of GSNO (1 mM) with Na_2_S (1 mM) and radical clock vinylcyclopropane (5 mM) in iP buffer at pH 7; (filled squares) in the presence of DH (5 mM), (filled circles) in the presence of IA (5 mM); (open squares) in the presence of DH and vinylcyclopropane, (open circles) in the presence of IA and vinylcyclopropane, and (filled diamond) in the presence of only vinylcyclopropane.

**Figure 11:**
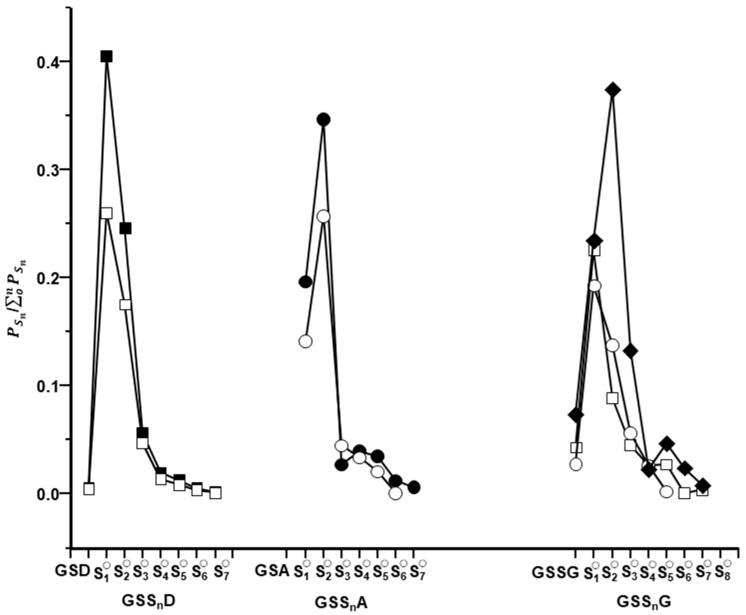
Relative distributions of GSS_n_-pent-2-ene (GSS_n_P), GSS_n_O-pent-2-ene (GSS_n_OP) determined from LCMS study of reaction of GSNO (1 mM) with Na_2_S (1 mM) and radical clock vinylcyclopropane (25 mM) in iP buffer at pH 7; (squares) in the presence of vinylcyclopropane, (up-pointing triangles) in the presence of DH and vinylcyclopropane, (circles) in the presence of IA and vinylcyclopropane, and (down-pointing triangles) in the presence of IA, DH, and vinylcyclopropane.

As can be seen in Figure 10, the presence of excess VCP decreases the maximum concentrations of the various GSX product families ca. 15–30% but has little effect on their relative distribution. Ring-opened VCP adducts are also seen, Figure 11, but at lower than one-tenth the intensity of polysulfane products. Therefore, we suggest that S-based radicals are indeed formed in these reactions but are short-lived, likely preceding the observed sulfenic acid GSOH. Furthermore, this agrees with our contention that polysulfanes are predominately generated via simple reactions between sulfenate and thiol, as seen in Equation (9).

## 3. Materials and Methods

Sodium sulfide nonahydrate, Na_2_S·(H_2_O)_9_, and reduced glutathione, GSH, were purchased from Acros Organics (Thermo Fisher Scientific, Newington, NH, USA). GSNO was synthesized following the literature method. Absorbance spectra were obtained using an Agilent 8453 spectrometer (Agilent, Santa Clara, CA, USA).

Liquid chromatography-high resolution mass spectrometry (LC-HRMS) samples were analyzed on an Accela liquid chromatograph coupled to an LTQ Orbitrap Discovery mass spectrometer (Thermo Electron, Bremen, Germany) using positive and negative electrospray ionization (+ESI/−ESI). Final extracts were diluted 100-fold into mobile phase and then injected (10 µL) into the LC system consisting of a 15 cm × 2.1 mm (5 µm, 80 Å) Extended-C18 column (Agilent Technologies, Palo Alto, CA, USA). A binary mobile phase gradient containing 0.1% (v/v) formic acid in water (A) and acetonitrile (B) was applied as follows: 97% A for 5 min, to 98% B in 30 min, held for 5 min, back to 97% A in 1 min, and equilibrated for 5 min at 97% A. Additional chromatographic parameters were as follows: Column temperature, 30 °C; flow rate, 350 µL/min. Full-scan accurate mass spectra (m/z range: 50–700) of eluting compounds were obtained at high resolution (30,000 FWHM) on the Orbitrap mass analyzer using internal calibration (accuracy of measurements <2 ppm) and processed using Xcalibur v.2.0.7 software (Thermo Fisher Scientific, NH, USA). Electrospray source conditions were: Sheath and auxiliary gas flow 50 and 5 arbitrary units (a.u.), respectively; heated capillary temperature 300 °C; electrospray voltage 4.5 kV for +ESI and 5.0 kV for −ESI; capillary voltage 43 V for +ESI and −43 V for −ESI; tube lens voltage 205 V for +ESI and −148 for −ESI.

### 3.1. General Protocol for Orbitrap LC-HRMS Analysis

All the reactions for LC/MS studies were carried out in a VMR ROBO autosampler vial (1.8 mL). Anaerobic reactions were either carried out in Vac Atmosphere glove box (OMNI-LAB) or in the Schlenk line under nitrogen atmospheres.

### 3.2. Reaction Protocols

All reactions were carried out in 50 mM sodium phosphate buffer at pH 7 unless otherwise described. For anaerobic reactions, an Omni-Lab glove box from VacAtmospheres Inc. (Hawthorne, CA, USA) or a Schlenk line was used to manipulate the anaerobic reaction solutions. Other reactions were carried out in septa sealed vials in a vented hood. Samples of stock solutions of IA in pH 7 iP buffer and DH in DMSO were added to reactions to achieve final concentrations as indicated in the text. *Warning: H_2_S is a toxic gas; all experiments were conducted to minimize its release and exposure to laboratory personnel*.

### 3.3. Relative Distribution Calculations

The relative distribution of the species under various reaction conditions was determined by calculating peak areas of selective ion chromatograms (SICs) of the individual species ($P_{S_{n}}$) extracted from the total ion chromatogram. The relative distribution for a group of species was obtained by dividing areas of the peak of an individual species by the total peak area of that group with sulfane sulfurs ranging from 0 to n, designated as ($P_{S_{n}}/\sum_{o}^{n}P_{S_{n}}$). The high sensitivity of the HDLC-HRMS obtains a wide range of relative distributions; therefore, the data are represented as the natural log of this fraction, $\ln(P_{S_{n}}/\sum_{o}^{n}P_{S_{n}})$.

### 3.4. Reactions of GSNO with H_2_S

Various equivalents of Na_2_S (0.25, 0.5, 1, 2.5, 5) were added to GSNO (1 mM) either in iP buffer pH 7 or in carbonate buffer, pH 10. The reaction solutions were allowed to stand for an hour before LC/MS analysis. For temperature-dependent studies the reaction solutions were incubated at 37 °C in Excella E24 incubator. For trapping reactions, the reagents DH and IA were added in situ or 1 h after reaction initiation, and reaction solutions analyzed by LC-HRMS. A number of control reactions were run at variable pH, temperature, and with the addition of elemental sulfur or thiosulfate, as described in the Supplemental materials (SI).

### 3.5. Radical Trapping Reactions

In a typical reaction, a septum-sealed vial (2 mL) containing a solution of GSNO (1 mM) in iP buffer at pH 7 was degassed by bubbling nitrogen in a Schlenk line for 3 min. Degassed samples of VCP in DMSO were added to a final concentration of 5 mM using a gastight syringe. For the trapping reactions, the degassed stock solutions of DH and/or IA were then added to a final concentration of 5 mM. The reaction was then initiated by adding degassed Na_2_S (1 mM) in iP buffer at pH 7 and allowed to stand for 1 h before LC-HRMS analysis.

## 4. Conclusions

To our knowledge, this work is the first characterization and comparative speciation of polysulfane species generated in the reaction of GSNO and H_2_S, a reaction fundamentally relevant to H_2_S signaling and vasodilation. As previously stated, polysulfides are implicated in cell signaling, but the mechanisms of their biosynthesis and activities are not well understood. To that end, we have used this reaction to interrogate various pathways in the formation of catenated polysulfur species in a biological milieu.

Our results indicate that once initiated, S-concatenation is quite facile. There is a clear predominance of shorter di- and tri-sulfanes among the reduced polysulfides, GS_n_H, which may have a kinetic rationale; Fukuto et al. have proposed that polysulfide equilibration leads preferentially to the trisulfide [48]. But there is no energetic or kinetic barrier to larger polysulfanes formation; the distributions fall statistically from penta- to nona-sulfanes, with no drop indicative of extrusion of elemental sulfur. In the same way, similar polysulfane (GSS_n_SG) distributions were observed in both aerobic or anaerobic reactions during in situ trapping reactions; the only variance was observed for aerobic reactions with delayed trapping, attributed to oxygenations of the polysulfides GSS_n_H, with dioxygen, confirmed by the lack of shorter polythiosulfonates species, e.g., GSS_n_O_3_H, with n = 0–2.

A key observation is that sulfenic acid derivatives GS_n_OH are generated under both aerobic and anaerobic conditions. Indeed, the simplest sulfenate GSOH (trapped as GSD) is also found in simple reduction reactions of GSNO, and thus it must represent a fundamental intermediate. Our previous report also suggested the reduction of GSNO by H_2_S occurs, as almost identical N-gas distributions are produced in GSNO reactions with H_2_S, dithiothreitol, and dithionite. Conversely, radical intermediates must be involved, as the radical ·NO is the major gaseous product in all these decompositions of GSNO. Using the unique trap VCP, we confirmed that S-based radicals were generated, but also showed that radicals did not directly influence polysulfide formation.

More telling were the competitive trapping experiments which showed sulfenic species precede higher polysulfide which precedes polysulfane formation, GS_n_OH → GSS_n_H → GSS_n_G. Thus, sulfenic species appear intimately involved in S-S bond formation. We contend that sulfur/sulfur catenation occurs mainly via reactions of the sulfides with sulfur-oxide species, and therefore a viable elemental step in the biosynthetic pathways of sulfur-concatenated species.

We have previously shown that small oxoacids of sulfur (SOS) like HSOH and HOSOH are generated from H_2_S oxidation [21], and thus are likely metabolites of H_2_S in vivo [22]. Here we suggest they are key to the formation of persulfides in the reaction of GSNO and H_2_S. The SOS species demonstrate reactivity and selectivity distinct from polysulfides and thus may function as independent H_2_S-derived signaling agents with unique biological effects.

## Supplementary Materials

The following are available online, S1. LCMS of oxidized polysulfides in MeOH with 1% formic acid mobile phase; S2. LC-MS/MS of GSS_n_G products; S3. Reaction of GSNO with H_2_S in carbonate buffer at pH 10; S4. Reaction of GSNO with H_2_S at different temperatures; S5. Reaction of GSNO with H_2_S gas; S6. Reactions of GSSG and GSH w/wo elemental Sulfur; S7. Comparison of the reaction of GSNO and H_2_S w/wo elemental Sulfur; S8. Reaction of GSH with sodium thiosulfate, Na_2_S_2_O_3;_ S9. Reaction of GSNO with H_2_S with and without NaS_2_O_3;_ S10. Mass spectra of reduced GSS_n_H identified in the reaction of GSNO with H_2_S; S11. LC-MS/MS of GSS_n_H products; S12. LC-MS/MS of GSS_n_SO_3_H products; S13. MS comparison of GSSH and GSO_2_H in aerobic reactions; S14. LC-MS/MS of GSS_n_SO_2_H products; S15. SIC and MS of GSD from GSNO reaction with ascorbic acid; S16. LC-MS/MS of GSS_n_NH_2_ products; S17. LC-MSMS of GSNS_n_A_2_ products
