# Correction: Kumar, M.R., et al. Characterization of Polysulfides, Polysulfanes, and Other Unique Species in the Reaction between GSNO and H_2_S. *Molecules* 2019, *24*, 3090

**DOI:** 10.3390/molecules24244610

**Published:** 2019-12-16

**Authors:** Murugaeson R Kumar, Patrick J Farmer

**Affiliations:** Department of Chemistry and Biochemistry, Baylor University, Waco, TX 76798, USA; Murugaeson_Kumar@baylor.edu

The authors wish to make the following corrections to this paper [1]: 

Figure 11 given in original publication was incorrectly a repeat of Figure 10. The correct Figure 11 is as follows:

This change does not affect the scientific outcomes as described in the text. The manuscript will be updated and the original will remain online on the article webpage. The authors would like to apologize for any inconvenience caused to the readers by these changes.

## Figures and Tables

**Figure 11:**
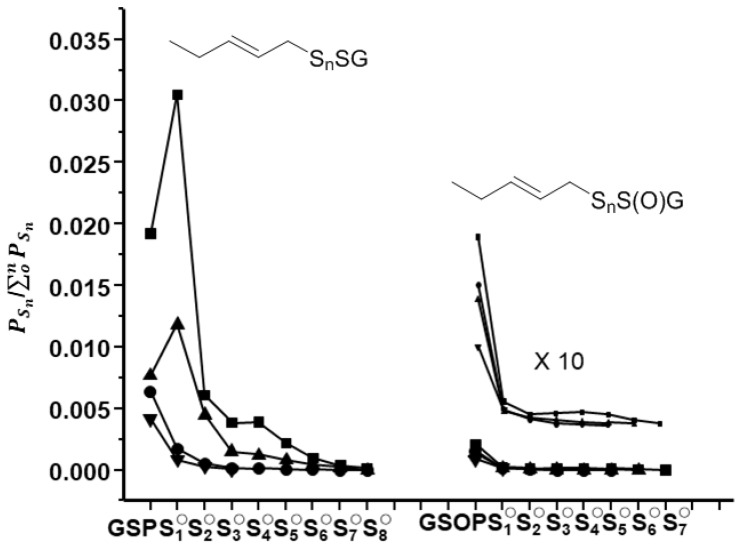
Relative distributions of GSSn-pent-2-ene (GSS_n_P) and GSS_n_O-pent-2-ene (GSS_n_OP) determined from LCMS study of reaction of GSNO (1 mM) with Na_2_S (1 mM) and radical clock vinylcyclopropane (25 mM) in iP buffer at pH 7; (squares) in the presence of vinylcyclopropane, (up-pointing triangles) in the presence of DH and vinylcyclopropane, (circles) in the presence of IA and vinylcyclopropane, (down-pointing triangles) in the presence of IA, DH, and vinylcyclopropane.
