# Supplementary material for: Characterization of Polysulfides, Polysulfanes, and Other Unique Species in the Reaction between GSNO and H2S
Source: Molecules. 2019 Aug 26;24(17):3090. doi: 10.3390/molecules24173090 (PMC6749520; doi:10.3390/molecules24173090)
Supplement: Supplementary file 1 [file molecules-24-03090-s001.pdf]

Characterization of polysulfides, polysulfanes and other unique species in the reaction between GSNO and H_2S

*Murugaeson R Kumar and Patrick J Farmer**

Department of Chemistry and Biochemistry, Baylor University, Waco, TX 76798

S1. LCMS of oxidized polysulfides in MeOH with 1% formic acid mobile phase

S2. LC-MS/MS of GSS_nG products

S3. Reaction of GSNO with H_2S in carbonate buffer at pH 10

S4. Reaction of GSNO with H_2S at different temperatures

S5. Reaction of GSNO with H_2S gas

S6. Reactions of GSSG and GSH w/wo elemental Sulfur

S7. Comparison of reaction of GSNO and H_2S w/wo elemental Sulfur

S8. Reaction **of** GSH with sodium thiosulfate, $Na_2S_2O_3$.

S9. Reaction of GSNO with H_2S with and without NaS_2O_3

S10. Mass spectra of reduced GSS_nH identified in the reaction of GSNO with H_2S.

S11. LC-MS/MS of GSS_nH products

S12. LC-MS/MS of GSS_nSO_3H products

S13. MS comparison of GSSH and GSO_2H in aerobic reactions

S14. LC-MS/MS of GSS_nSO_2H products

S15. LC-MS/MS of GSS_nNH_2 products

S16. LC-MSMS of $GSNS_nA_2$ products

S1. LCMS of oxidized polysulfides in MeOH with 1 % formic acid mobile phase

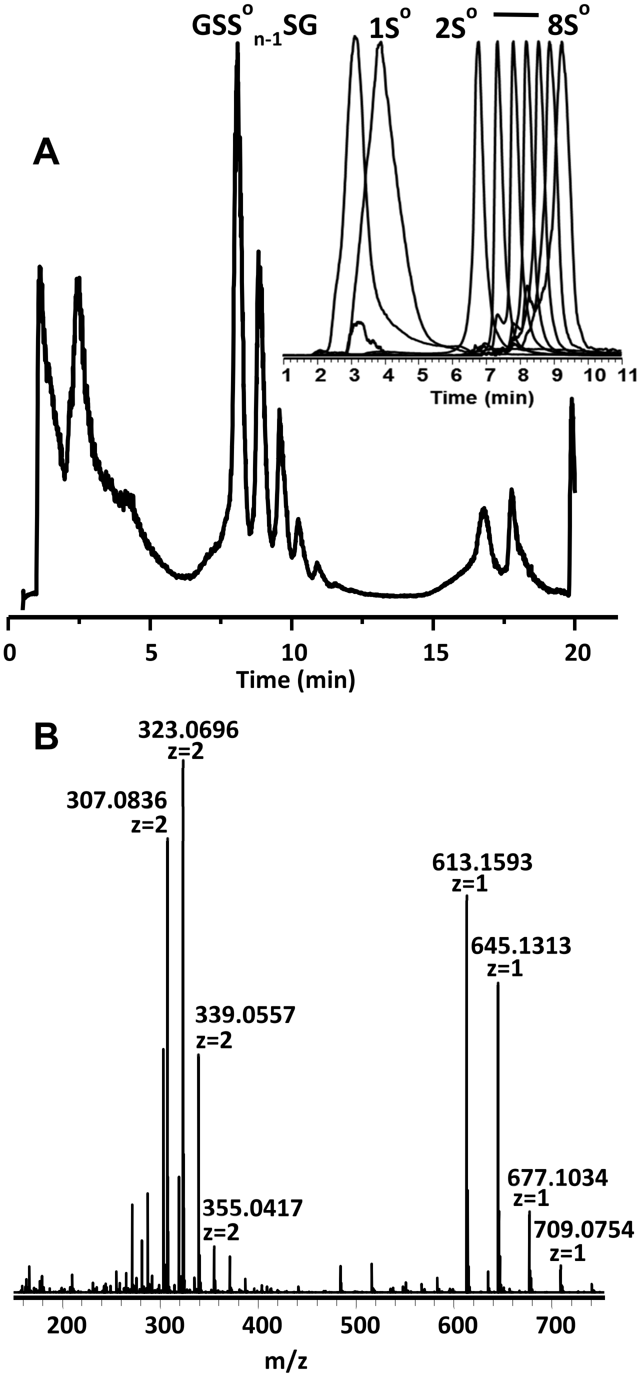

Figure S1. LC-HRMS analysis of oxidized glutathione polysulfane species formed in a 1:5 reaction of GSNO with Na_2S at pH 7, with 0.1% formic acid-methanol as carrier stream. A) The total ion LC spectra. (inset) Normalized selective ion chromatograms (SICs) showing a series of GSS_nSG polysulfanes, n = 0-8, and the separation times of each within the overall LC spectra. (B) Region of the total ion mass spectra showing peaks assigned as shown, to dications.

S2. LC-MS/MS of GSS_nG products.

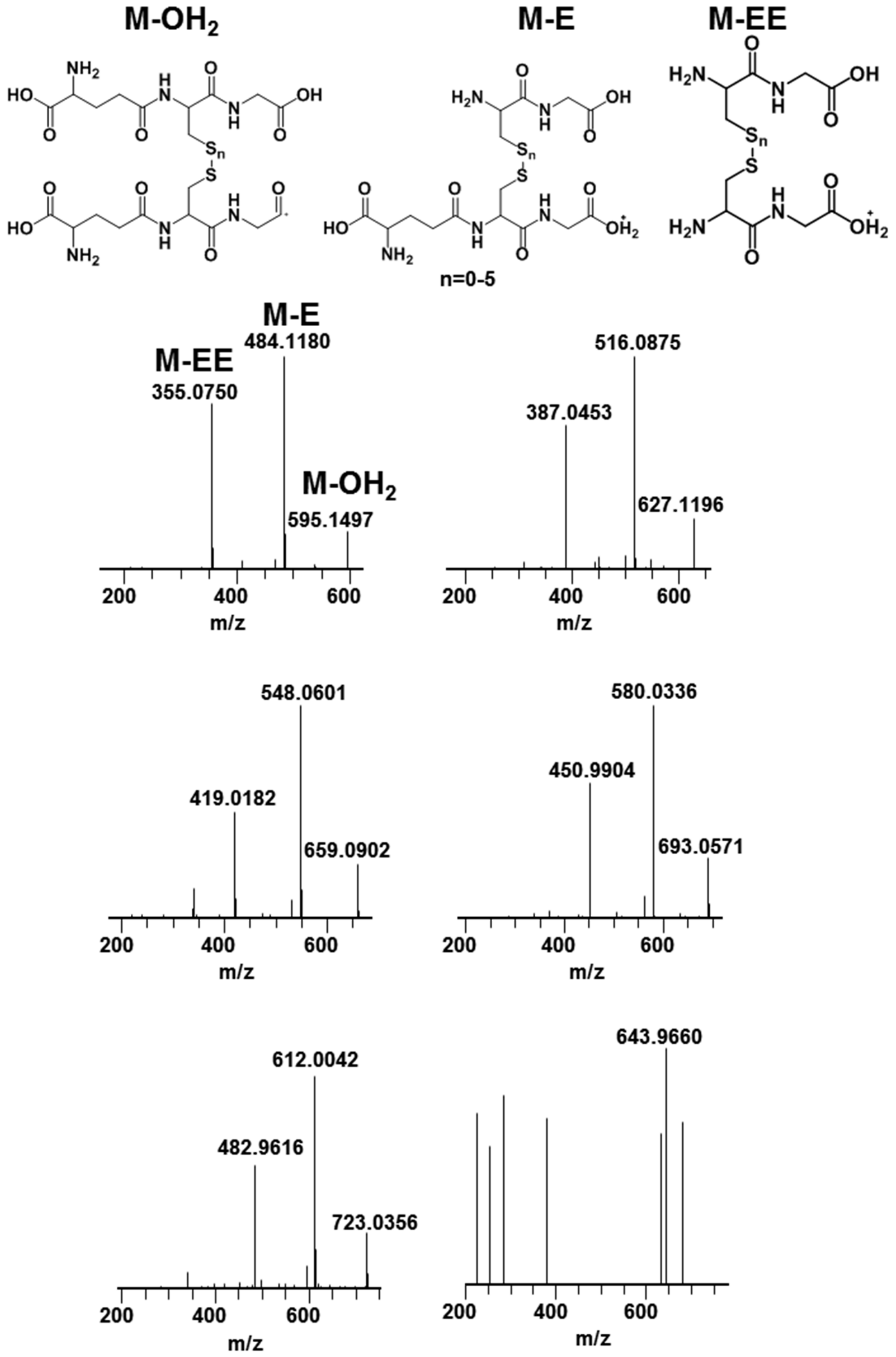

Figure S2. LCMS MS/MS study of glutathione polysulfides (oxidized) formed in the reaction of GSNO 1 mM) with Na_2S (5 mM) in iP buffer at pH 7.

S3. Reaction of GSNO with H_2S in carbonate buffer at pH 10.

High pH reactions of GSNO with H_2S. The reaction solutions were allowed to stand for an hour before LC/MS analysis. For temperature dependent studies the reaction solutions were incubated at 37 ^{O}C in Excella E24 incubator.

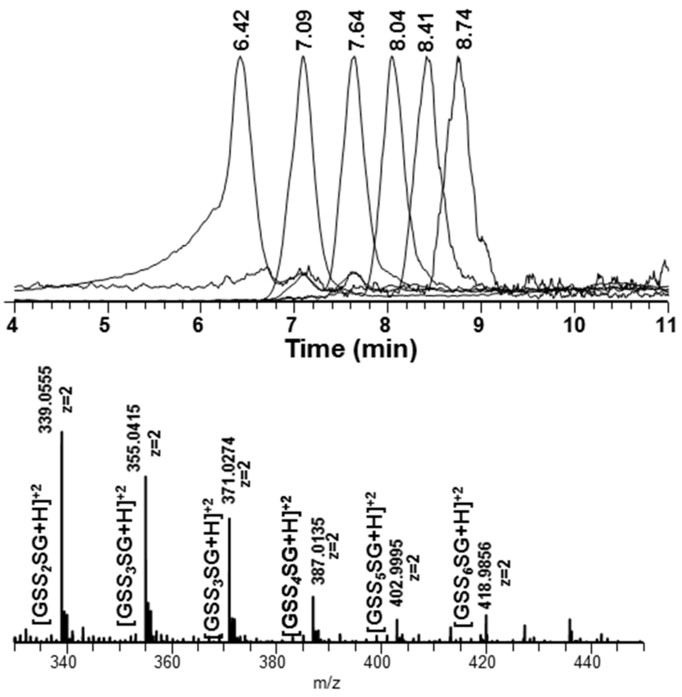

Figure S3. Reactions at high pH. Orbitrap LCMS study of reaction of GSNO (1 mM) with Na_2S (5 mM) in carbonate buffer at pH 10. (Top) SIC of glutathione polysulfides (oxidized) species and (Bottom) their corresponding mass spectra.

S4. Reaction of GSNO with H_2S at different temperatures

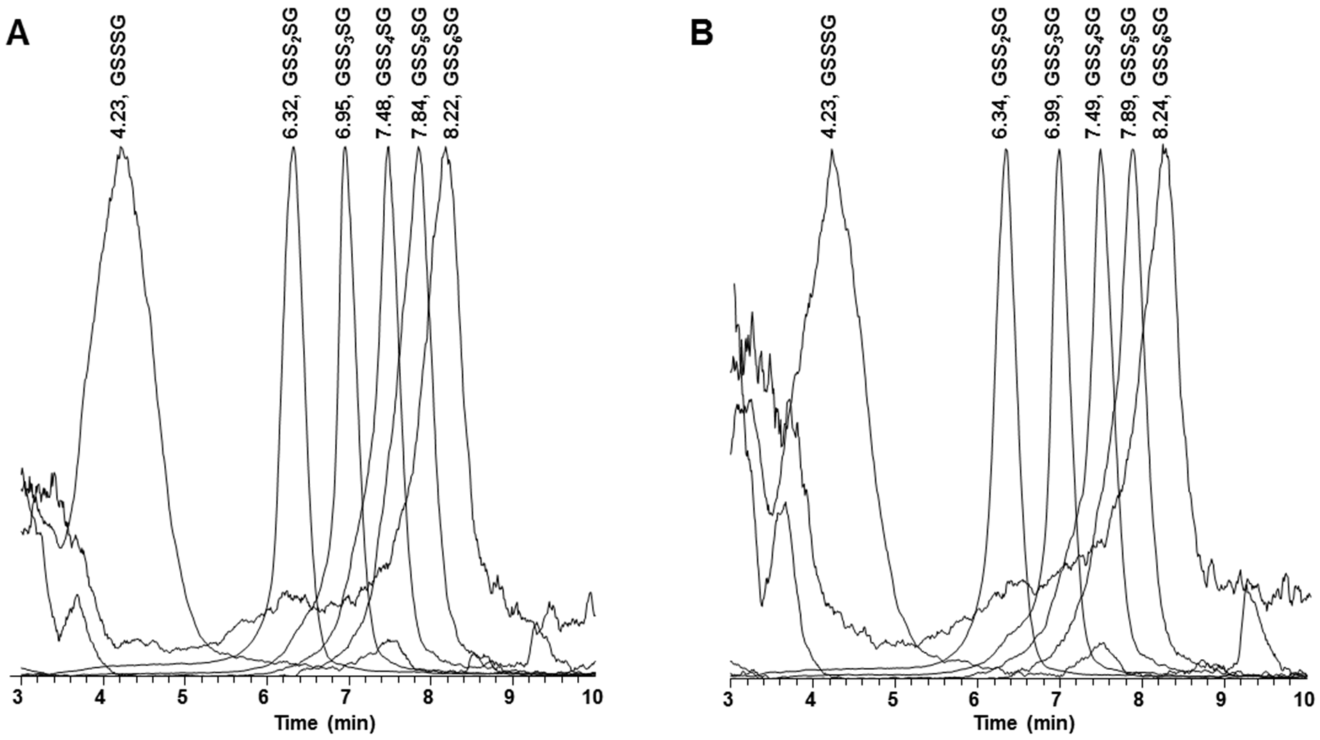

Figure S4. SIC of glutathione polysulfides obtained from the analysis of LCMS of reaction of GSNO (1 mM) with Na_2S (5 mM) in iP buffer at pH 7. (A) 25 °C and (B) 35 °C.

S5. Reaction of GSNO with H_2S gas

Reaction of GSNO or GSSG with $H_2S(g)$. As a control reaction. gaseous H_2S was generated by addition of concentrated HCl to Na_2S under inert N_2 purge which was bubbled directly through an aqueous solution of GSNO or GSSG in iP buffer pH 7. The reaction solutions were allowed to stand for an hour before LC/MS analysis.

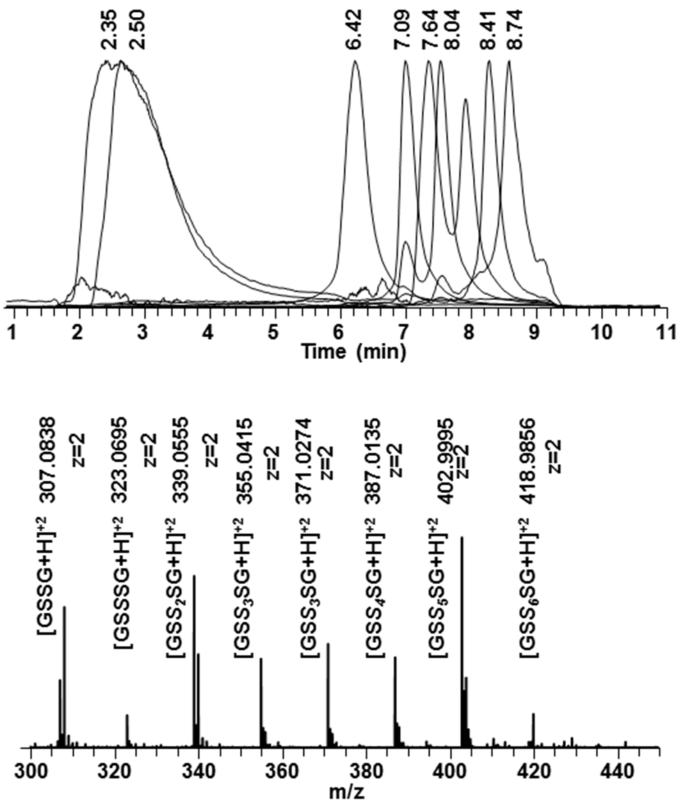

Figure S5. Orbitrap LCMS study of reaction of GSNO (1mM) with H_2S (gas) generated from Na_2S-HCl mixture in iP buffer at pH 7. (Top) SIC of glutathione polysulfides (oxidized) species and (Bottom) their corresponding mass spectra.

S6. Reactions of GSSG and GSH with elemental Sulfur.

Reactions with elemental Sulfur(s). To reaction solutions of GSH (1 mM) or GSSG (1 mM) were added elemental sulfur (solid) in iP buffer, pH 7 and allowed to stand for 24 h.

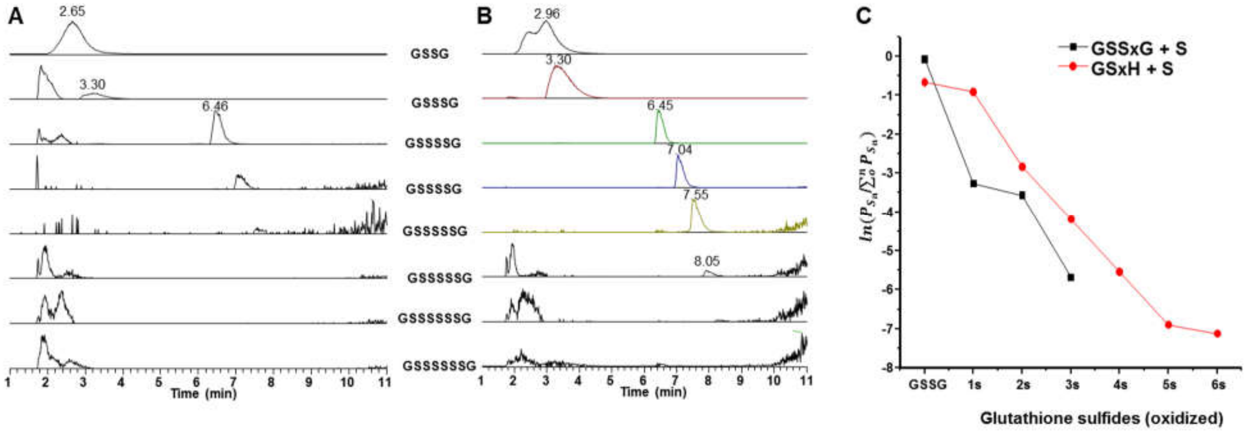

Figure 6. SICs of oxidized glutathione polysulfides (GSS$_x$SG) from the Orbitrap LCMS analysis of reaction of (A) 1 mM of GSSG, (B) 1 mM of GSH with excess S in iP buffer at pH 7 and (C) relative distribution of the glutathione polysulfide species.

S7. Comparison of reaction of GSNO with H_2S with and without the presence of elemental Sulfur.

Reactions of GSNO/H_2S in presence and absence of S(s). To the reaction solutions of GSNO (1 mM) and Na_2S (5 mM), were added elemental sulfur (solid) in iP buffer, pH 7 and allowed to stand for 24 h.

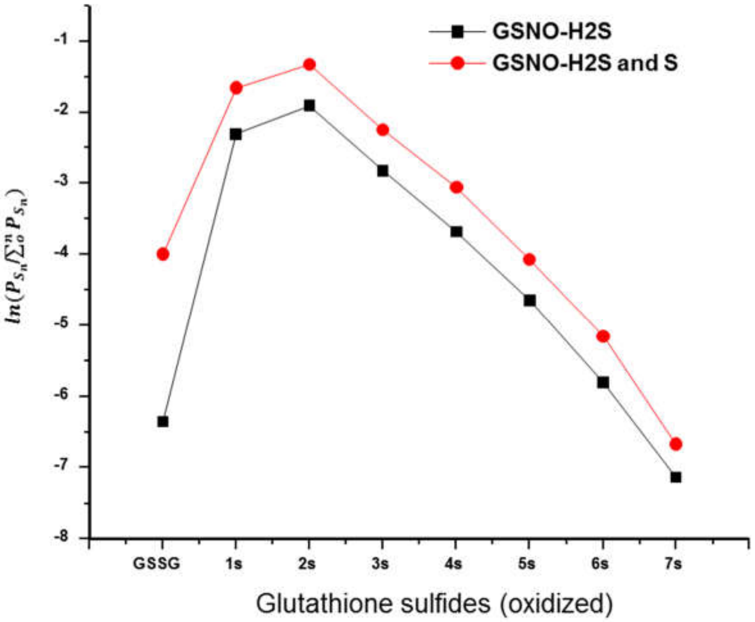

Figure S7. Relative distribution of the glutathione polysulfide species calculated in the presence (red) and absence (black) of S with GSNO (1 mM) and Na_2S (5 mM) in iP buffer at pH 7.

S8. Reaction **of** GSH with sodium thiosulfate, $Na_2S_2O_3$.

Reactions of sulfite and thiosulfate with GSH. 5 mM of GSH was mixed either with 10-fold excess Na_2SO_3 or $Na_2S_2O_3$ in iP buffer pH 7. The reaction solutions were allowed to stand overnight before LC/MS analysis.

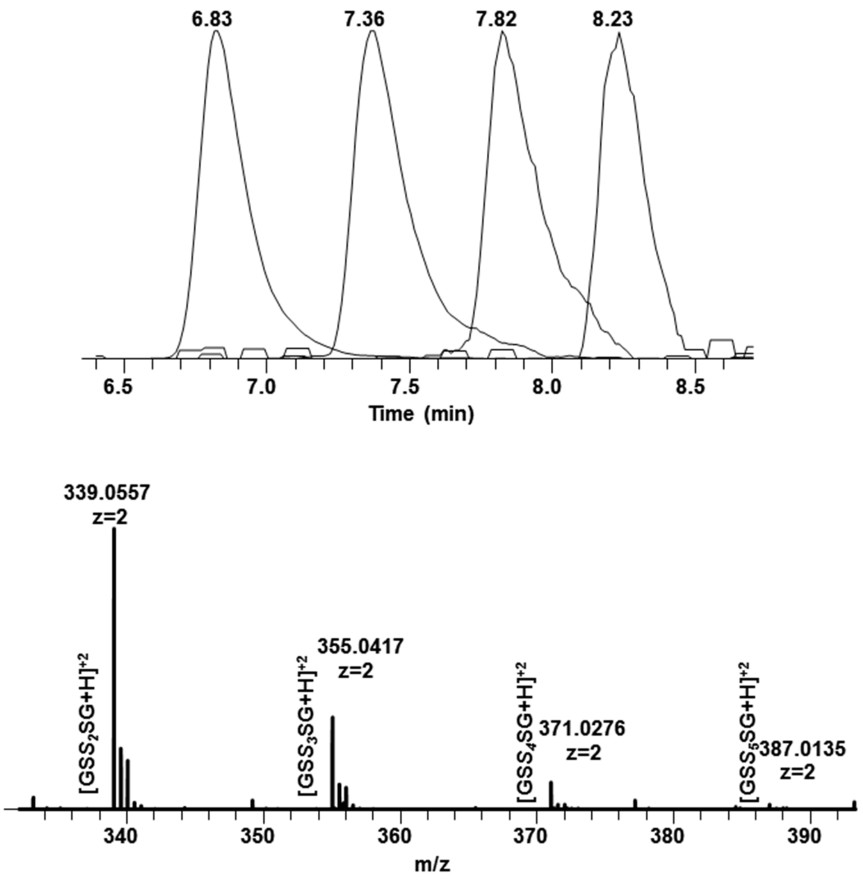

Figure S8. Orbitrap LCMS study of reaction of GSH (1 mM) with $Na_2S_2O_3$ (5 mM) in iP buffer at pH 7. Top: SIC of glutathione polysulfides (oxidized) species. Bottom: their corresponding mass spectra.

S9. Reaction of GSNO with H_2S with and without NaS_2O_3.

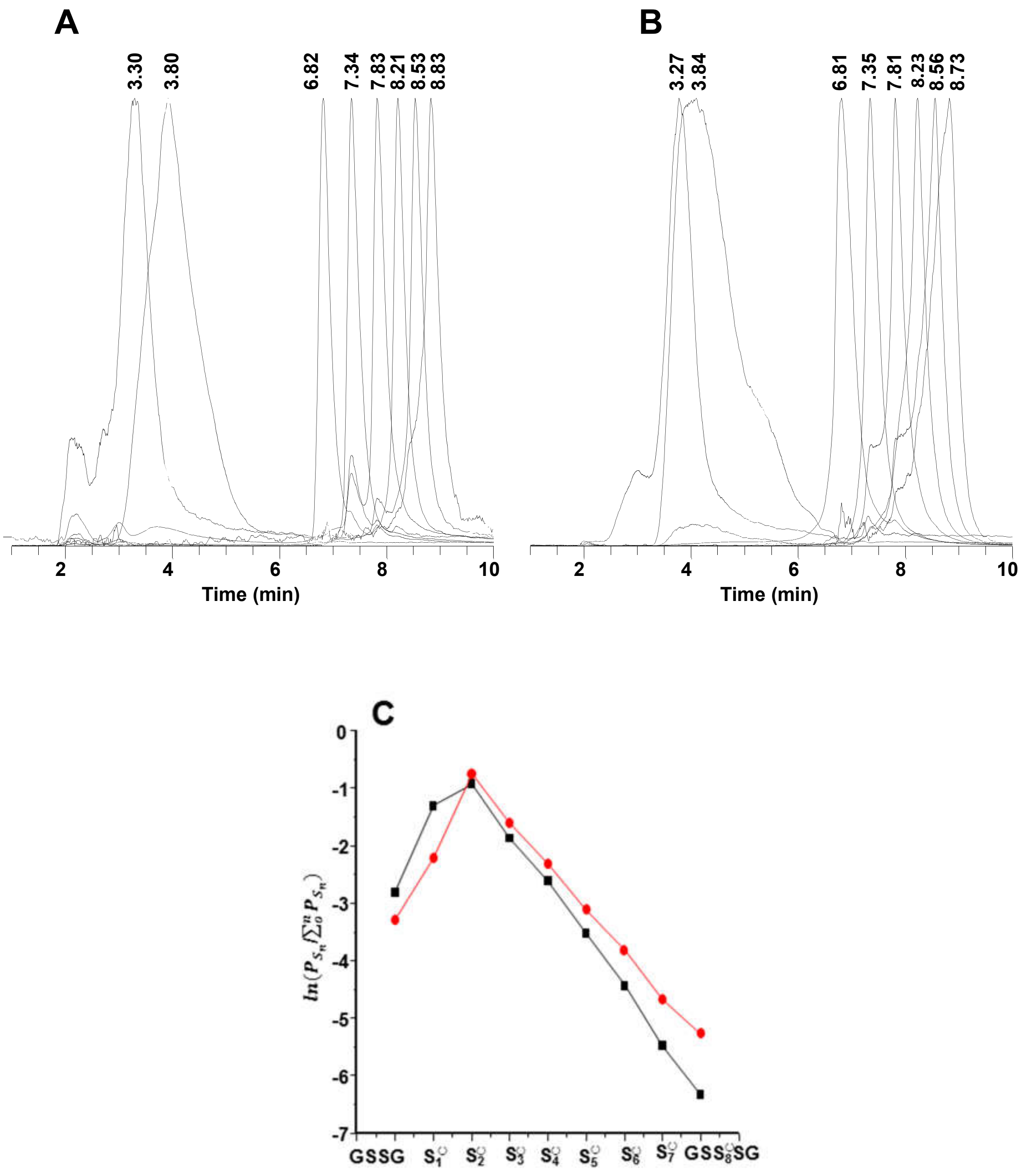

Figure S9. Orbitrap LCMS study of reaction of GSNO (1 mM) with Na_2S (5 mM) in iP buffer at pH 7, in the (**A**) absence, (**B**) presence of $Na_2S_2O_3$ (5 mM) and their relative distributions of GSS_nSG products (**C**); $Na_2S_2O_3$ absence (squares) and $Na_2S_2O_3$ presence (circles).

S10. Mass spectra of reduced GSS_nH identified in the reaction of GSNO with H_2S.

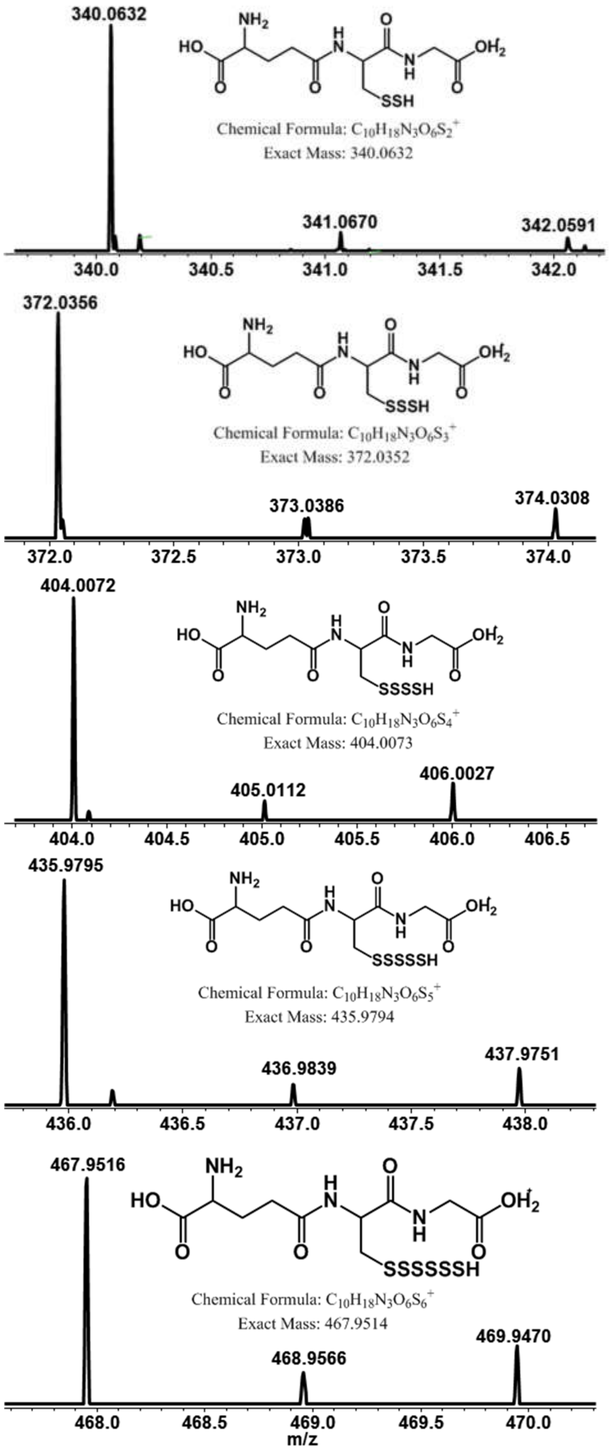

Figure S10. Orbitrap MS glutathione polysulfides (reduced) formed in the reaction of GSNO (1 mM) with Na_2S (5 mM) in iP buffer at pH 7.

S11. LC-MS/MS of GSS_nH products

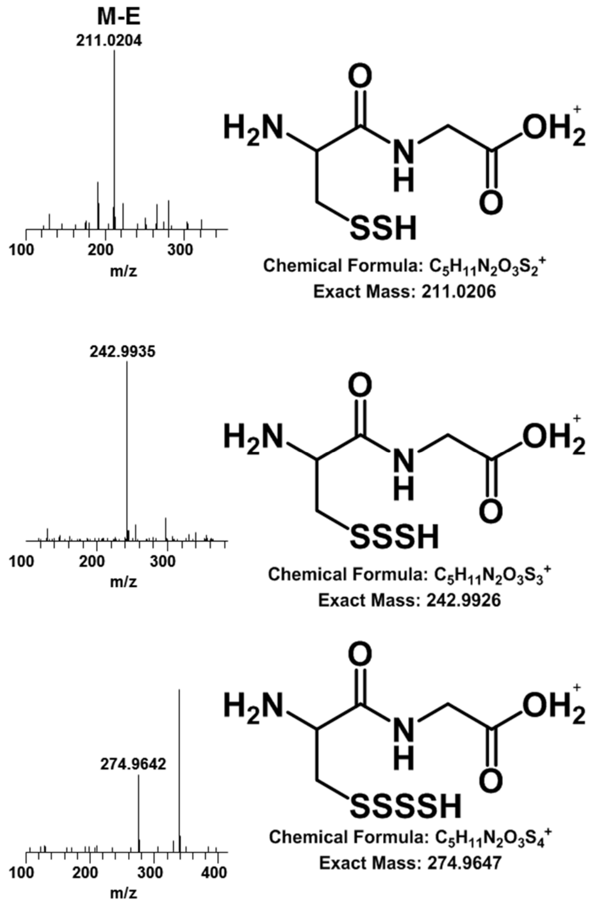

Figure S11. Orbitrap LCMS MS/MS study of glutathione polysulfides (reduced) formed in the reaction of GSNO (1 mM) with Na_2S (5 mM) in iP buffer at pH 7.

S12. LC-MS/MS of GSS_nSO_3H product

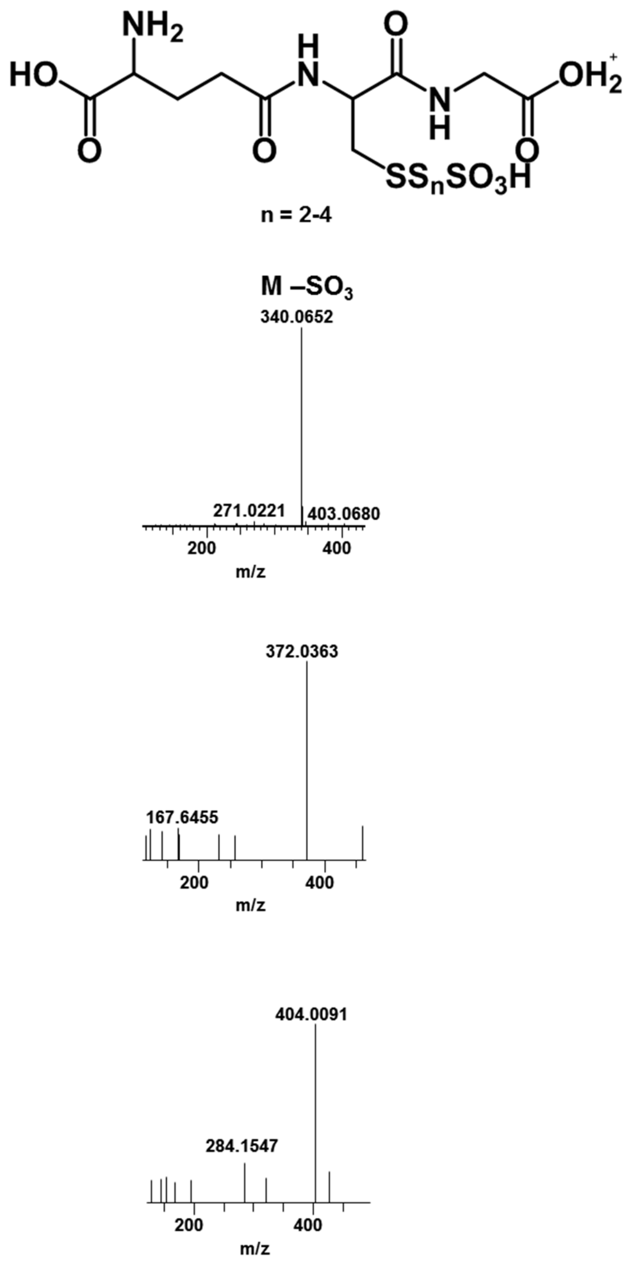

Figure S12. Orbitrap LCMS MS/MS study of glutathione polythiosulfonates formed in the reaction of GSNO (1 mM) with Na_2S (5 mM) in iP buffer at pH 7.

S13. MS comparison of GSSH and GSO_2H in aerobic reactions

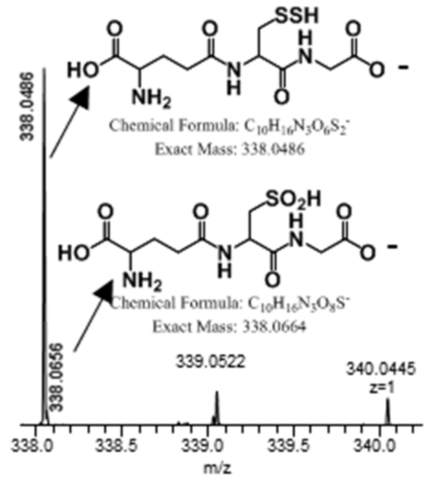

Figure S13. LCMS study in the negative ion mode of GSSH and GSO_2H and glutathione sulfinic acid formed in the reaction of GSNO (1 mM) with Na_2S (5 mM) in iP buffer at pH 7.

S14. LC-MS/MS of GSS_nSO_2H product

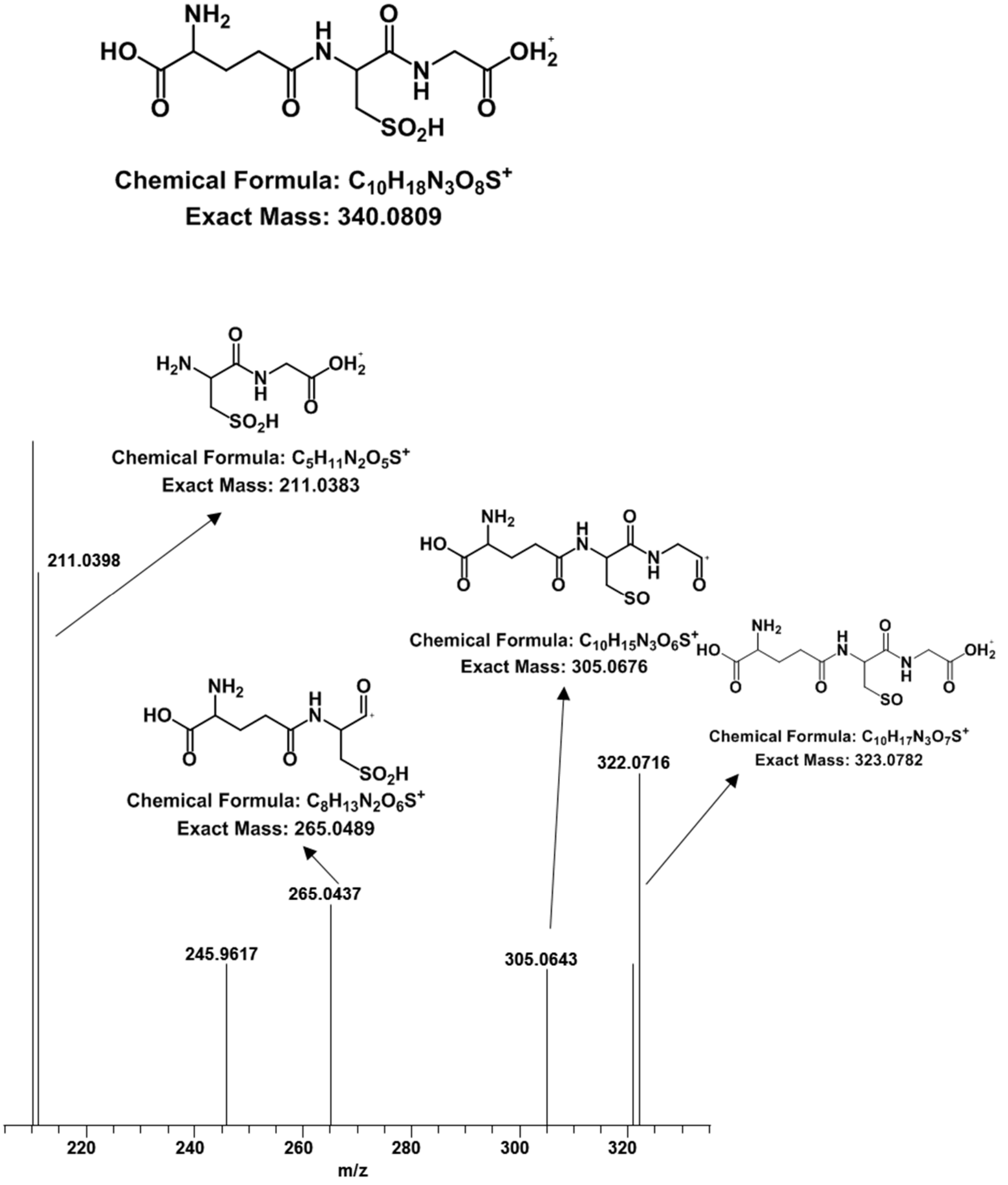

Figure S14. Orbitrap LCMS MS/MS study of glutathione sulfinic acid formed in the reaction of GSNO (1 mM) with Na_2S (5 mM) in iP buffer at pH 7.

S15. LC-MS/MS of GSS_nNH_2 product

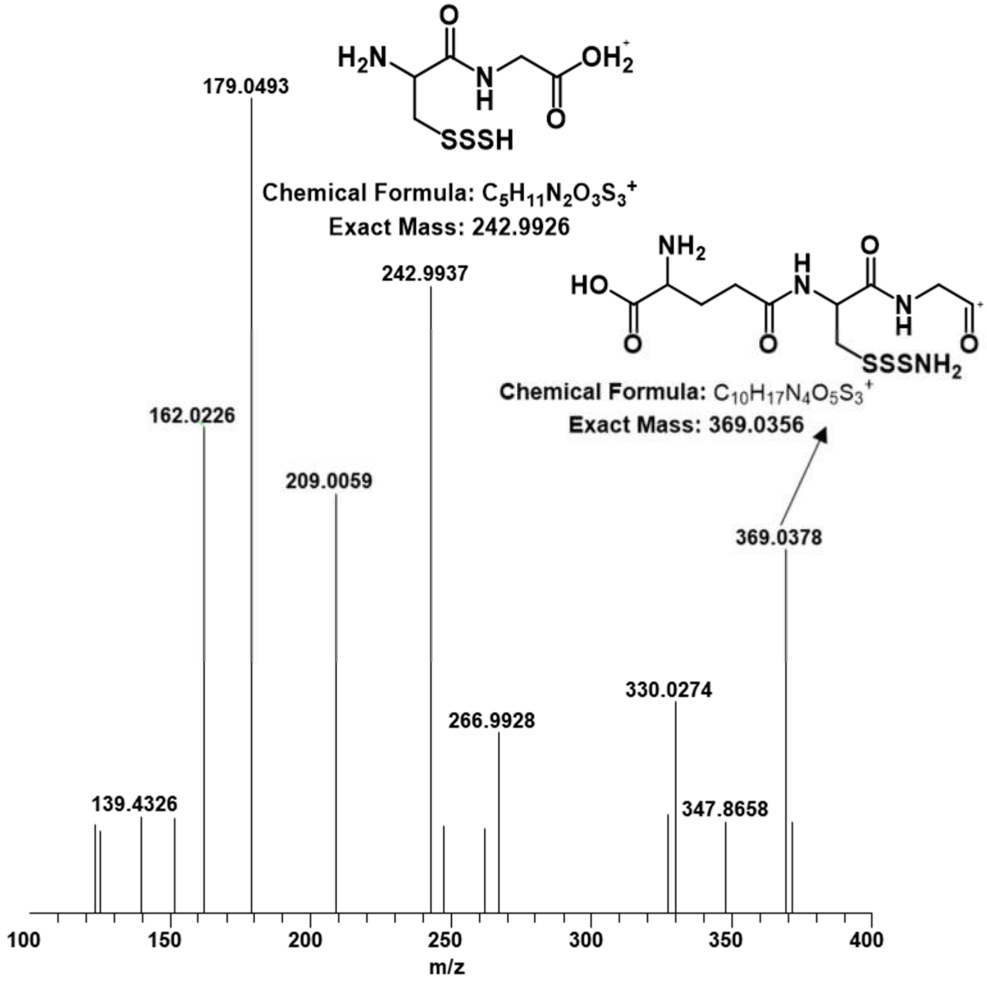

Figure S15. Orbitrap LCMS MS/MS study of GSS_nNH_2 products formed in the reaction of GSNO (1 mM) with Na_2S (5 mM) in iP buffer at pH 7.

S16. LC-MS/MS of GSS_nNA_2 product

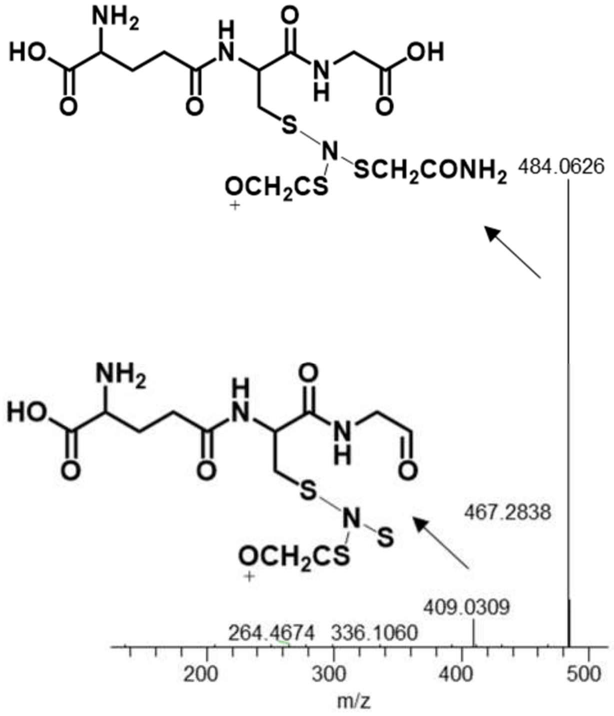

Figure S16. Orbitrap LCMS MS/MS study of $GSNS_nA_2$ products formed in the reaction of GSNO (1 mM) with Na_2S (5 mM) and IA (5 mM) in iP buffer at pH **7.**
